# Usability and understandability of a web-based medical communication aid for patients with ankylosing spondylitis in South Korea: A mixed-methods study

**DOI:** 10.1097/MD.0000000000033430

**Published:** 2022-04-07

**Authors:** Sang-Hoon Lee, YoungJu Park, Chan-Bum Choi, Yong-Gil Kim, Jung-Ae Kim, Hoon-Suk Cha

**Affiliations:** a Department of Rheumatology, Kyung Hee University Hospital at Gangdong, School of Medicine, Kyung Hee University, Seoul, South Korea; b Medical Affairs, Janssen Korea, Seoul, South Korea; c Department of Rheumatology, Hanyang University Hospital for Rheumatic Diseases, Seoul, South Korea; d Division of Rheumatology, Department of Internal Medicine, University of Ulsan College of Medicine, Seoul, South Korea; e Real World Solutions, IQVIA Korea, Seoul, South Korea; f Department of Medicine, Samsung Medical Center, Sungkyunkwan University School of Medicine, Seoul, South Korea.

**Keywords:** ankylosing spondylitis, medical communication aid, patient decision-making tool, shared decision-making

## Abstract

Ankylosing spondylitis (AS) is a chronic inflammatory arthritis which causes potentially debilitating pain and loss of mobility. Biologics represent a highly effective treatment option in AS. Nonetheless, the choice of biologics often involves complex decision-making. A web-based medical communication aid (MCA) was designed to support information exchange and shared decision-making process between physicians and biologics naïve AS patients. This study aimed to assess the usability of the MCA prototype and the understandability of the MCA contents among rheumatologists and AS patients in South Korea. This was a cross-sectional study using a mixed-methods approach. Treating rheumatologists from major hospitals and their AS patients were recruited in this study. Participants navigated through the MCA and provided feedbacks, guided by interviewers using the think-aloud (TA) method. Participants were then asked to complete a set of surveys. The qualitative and quantitative data were analyzed to determine the usability of the MCA prototype and the understandability of the MCA contents. The MCA prototype received above average rating for usability and high rating for the understandability of its contents. Additionally, participants rated that the quality of information presented in the MCA as high. Analysis of the qualitative data highlighted three key aspects of the MCA; the usefulness of the MCA, the need to present concise and relevant content; and the importance of an intuitively designed tool. Overall, participants found the MCA to be potentially valuable in supporting the current unmet needs in clinical care and had expressed a willingness to use the MCA. The MCA had great potential in supporting shared decision-making by improving patients’ knowledge on disease and treatment options, as well as clarifying patients’ personal preferences and values in the management of AS.

## 1. Introduction

Ankylosing spondylitis (AS) is a chronic inflammatory arthritis which primarily affects the axial skeleton.^[1]^ The main clinical symptoms of patients with AS are pain and stiffness of the lower back.^[2,3]^ AS affects men more often than women, with early disease onset at an age younger than 45 years.^[1]^ The treatment of AS is tailored based on several factors; the manifestations of the disease at presentation, severity of symptoms and patient preferences. A combination of non-pharmacological and pharmacological treatments is typically used to alleviate pain and physical impairment, and to improve patient’s quality of life.^[4]^ Non-pharmacological treatment includes physical therapy, exercise and patient education while pharmacological treatment includes medications such as non-steroidal anti-inflammatory drugs (NSAIDs), disease-modifying anti-rheumatic drugs, and biologics.^[4,5]^

Biologics such as the tumour necrosis factor-alpha inhibitor and interleukin-17 inhibitors have shown to be effective in AS, with a high response rate in patients not responding to NSAIDs.^[6,7]^ However, concerns such as cost and adverse events associated with the use of these biologics may impact adherence to and persistence with therapy.^[8,9]^ With an increasing number of biologics available, the decision-making in selecting the most appropriate agent becomes even more complicated. The unique benefits and side effects of each biologic needs to be carefully considered, along with patient preferences.

Healthcare professionals play an important role in providing information and advice to AS patients.^[10]^ Patient education aims to support and strengthen patient’s self-efficacy and adherence to treatment.^[11]^ With an increased knowledge and understanding of AS and its management, patients will be able to participate more actively in decision makings regarding their health. The incorporation of patient’s preference in decision-makings promotes adherence to treatment, satisfaction with care and thus positively influence health outcomes.^[12–14]^ Despite the importance of interactive communication between physicians and patients, the information exchange between physicians and patients are limited due to given high patient load and limited resources in real world clinical settings. Therefore, we developed an easily accessible, web-based medical communication aid (MCA) to facilitate the information exchange process between physicians and biologics naïve AS patients who are considering biologics treatment. The MCA was designed to support shared decision-makings between physicians and AS patients by enabling better quality decisions through improved understanding of patients’ values and assessment of treatment risk. The development of the MCA prototype was guided by findings from a systematic literature review on the potential attributes considered in shared decision-making process between AS patient and physician. Additionally, we leveraged results from surveys and in-depth interviews with physician and AS patients to understand the limitations with the current clinical care and communication process. The MCA provides evidence-based information on 29 topics related to disease progression and available treatment options which were identified from the in-depth interview. Furthermore, the MCA visualizes the information that help patients better understand and interpret that information.

The objectives of this study were: to assess the usability of the MCA prototype and identify its limitation from the patient and physician’s perspective; and to evaluate the understandability of MCA contents. The results from this study will be used to further refine the MCA prototype.

## 2. Methods

### 2.1. Study design

This was a cross-sectional study using a mixed-methods approach. We conducted surveys and semi-structured interviews, with embedded think-aloud (TA) method to assess the usability of the MCA prototype and the understandability of the MCA contents among physicians and AS patients.

#### 2.1.1. Participants and recruitment.

We recruited ten rheumatologists and twenty of their AS patients from November 20, 2018 to December 20, 2018. This sample size was deemed optimal in identifying at least 80% of usability problems.^[15–17]^ Eligible physicians who worked at major hospitals in Korea were invited via telephone or email to participate in this study. Physicians were eligible to participate if they; were rheumatologists with experience in treating AS patient; were working at tertiary or general hospitals in Korea; and could provide informed consent. Using snowball sampling, recruited physicians will be asked to refer other physicians and patients for this study. We included patients if they; were at least 18 years of age; had a diagnosis of AS; were biologics naïve; could read or write Korean; and could provide informed consent. All study participants provided informed consent. The study was approved by the Korea National Institute for Bioethics Policy designated by the Korea Ministry of Health and Welfare (P01-201809-23-009).

#### 2.1.2. Study procedure.

Each interview was conducted in a meeting room or clinic waiting room and lasted approximately one hour. Interviews were conducted by research staffs who were not involved in the development of the MCA prototype. Prior to beginning the session, research staffs provided explanation on the general purpose of the testing, the approximate time required to complete the test, and obtained consent from study participants. The interviews were audio-recorded with consent from all study participants. Participants were then introduced to the web-based MCA prototype on a tablet computer. The TA approach would be followed as the participants moved through the MCA and complete a set of tasks. At the end of the testing, participants were asked to complete the relevant surveys; System Usability Scale (SUS), Patient Education Materials Assessment Tool (PEMAT), Information Quality Assessment. The results from these surveys would be used to determine the usability and understandability of the MCA prototype. Additionally, we collected information on the characteristics of study participants. Patients provided information on their socio-demographics, disease and medical history, familiarity with the internet and health literacy while physicians provided demographics and level of experience in AS management. The health literacy of patients was evaluated by using a shorten from of the Korean Health Literacy Scale, which consists of 7 comprehension and numeracy questions and 5 health-related questions.^[18]^

### 2.2. Measures

#### 2.2.1. Quantitative prototype testing.

##### 1.2.2.1. System usability scale.

The SUS was developed in 1996 as a “quick and dirty” survey scale to assess the usability of a given product or service. The SUS has proven itself a valuable and robust tool in assessing the quality of a broad spectrum of user interfaces. The SUS consists of 10 items with a 5-point Likert scale that ranges from strongly disagree to strongly agree. The overall value of SUS is calculated by multiplying the sum of scores by 2.5 and the SUS scores have a range of 0 to 100. A score below 50 would be considered unacceptable whereas scores in 70s and 80s are considered marginal at best.^[19,20]^ In this study, the SUS was measured from both the patients’ and physicians’ perspectives, using the SUS questionnaire translated and validated by Cho et al^[21]^ An approval for the use of questionnaire was obtained from the original authors.

##### 1.2.2.2. Patient Education Materials Assessment Tool.

The PEMAT is a systematic method to evaluate the understandability and actionability of patient education materials. This assessment tool was designed to be completed by professionals including health care providers, health librarians and others tasked with providing high-quality materials to patients or consumers. In this study, we used the PEMAT to assess understandability from the physicians’ perspective. Eleven items measuring understandability were adapted from the PEMAT-A/V version designed for audiovisual materials. Each item had the response options “Disagree (0 point)” or “Agree (1 point).” The PEMAT scores range from 0 to 100 and the percentage score on the PEMAT is calculated by multiplying the PEMAT understandability score by 100. A material with a PEMAT score of 70% and above was considered understandable.^[22]^ An approval for the use of questionnaire was obtained from the original author.

##### 1.3.2.2. Information Quality Assessment.

We assessed the quality of information presented in the MCA using selected attributes from a previous study.^[23]^ The attributes assessed from the patients’ perspective were appropriateness in the amount of text, believability, completeness, concise representation and understandability. The physicians provided assessments on the amount of text, believability, completeness, concise representation, objectivity, relevancy. Patients and physicians were given the option of “Disagree” or “Agree” to each attribute.

#### 2.2.2. Qualitative prototype testing.

Semi-structured interviews were conducted, with embedded TA method to evaluate the usability of the MCA prototype and the understandability of the MCA contents. The TA method allowed us to obtain further insights on the study participants’ experience while using the MCA prototype. TA is widely used to collect data in usability testing of software, interfaces, websites and instructional documents.^[24,25]^ During the interview, participants were encouraged to verbalize their thoughts and feelings while navigating through the MCA contents and completing a set of tasks using the MCA prototype. The research staff would prompt participants to elaborate what they were looking at, thinking or feeling when completing the tasks. The most commonly used prompts included “What are you thinking?” or “Can you try to say what is on your mind while doing the tasks or reading the contents?”

### 2.3. Statistics

Descriptive analyses were used to assess participant characteristics and scores from the SUS, PEMAT and Information quality assessment. All scores were calculated using standard methodology as recommended in the tool manuals. For the qualitative analysis, conversations during the interviews were transcribed and analyzed. Researchers read the transcripts several times to familiarize themselves with the data. Common themes or patterns were identified from the participants’ comments on the MCA prototype. De-identified quotes were extracted from the audio recordings.

## 3. Results

### 3.1. Participant’s characteristics

In total, 10 rheumatologists and 20 of their AS patients participated in this study. The participants’ characteristics are shown in Table 1. Patients were predominantly young males with reported disease durations of less than 1 year to more than 10 years. Majority of patients were on treatment with NSAIDs, with good overall health literacy. All physicians were rheumatologists, mainly practicing at general hospitals with an average clinical experience of 14.8 years.

**Table 1:** Demographic characteristics of participants.

Characteristic	Patients (n = 20)	Physicians (n = 10)
Age, mean (SD)	35.9 (11.5)	46.1 (4.6)
Age, n (%)		
20s	7 (35.0)	
30s	7 (35.0)	1 (20.0)
40s	2 (10.0)	7 (70.0)
50s	4 (20.0)	2 (20.0)
Male, n (%)	18 (90.0)	8 (80.0)
Current visiting/working hospital		
Tertiary hospital	7 (35.0)	3 (30.0)
General hospital	13 (65.0)	7 (70.0)
Current treatment, n (%)		
DMARDs	1 (5.0)	N/A
NSAIDs	15 (75.0)	N/A
NSAIDs + DMARDs	4 (20.0)	N/A
Duration of AS (yr), n (%)		N/A
<1	3 (15.0)	N/A
1–5	5 (25.0)	N/A
5–10	5 (25.0)	N/A
>10	7 (35.0)	N/A
Literacy score, mean (SD)	11.6 (0.8)	N/A

Values are presented as mean (SD) or n (%).

AS = ankylosing spondylitis, DMARDs = disease-modifying anti-rheumatic drugs, N/A = not applicable, NSAIDs = non-steroidal anti-inflammatory drugs, SD = standard deviation.

### 3.2. Quantitative prototype testing

#### 3.2.1. Usability.

**Figure 1.**
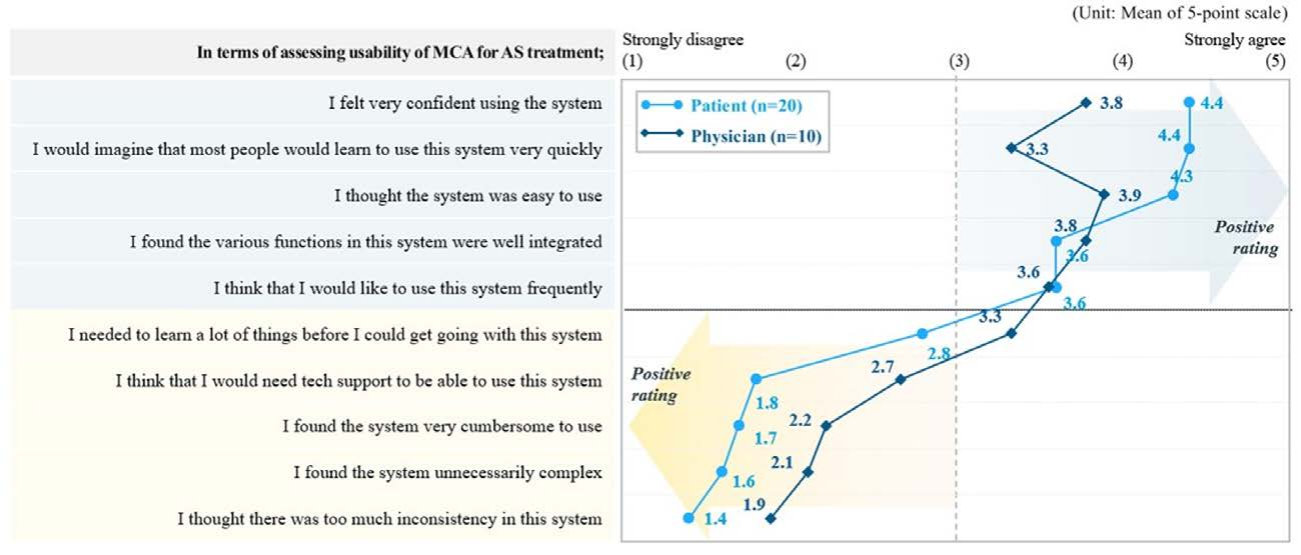
Results on usability assessment using System usability scale (SUS): level of agreement. Values are presented as mean (SD). AS = ankylosing spondylitis, SD = standard deviation.

The resulting mean scores from the SUS were 77.8 (SD = 14.02) for patients; and 65.3 (SD = 23.66) for physicians (Table 2; Fig. 1). The MCA tool was found to be well-structured and useable in providing information to patients and physicians. For patients, MCA had a user-friendly interface (e.g., text, image, animation) that made it easy to use. While patients were more receptive of the MCA, physicians found the tool to be cumbersome. This was evident through the lower mean SUS score for physicians *vs.* patients. The physicians’ main concern was on the practicality of the patient survey function of the MCA. The survey function was designed to support the physicians in identifying treatment attributes which were important to the patients such as costs and side effects. Additionally, physicians felt that there might be challenges in navigating through information without technical support on their first attempts. Some minor technical and illustrative changes may be needed to further improve the usability of the tool.

**Table 2:** Results on usability assessment using SUS: level of agreement.

SUS items	SUS score (mean, SD)	
	Patients (n = 20)	Physicians (n = 10)
I felt very confident using the system	4.45 (0.86)	3.78 (0.63)
I would imagine that most people would learn to use this system very quickly	4.45 (0.74)	3.33 (0.94)
I thought the system was easy to use	4.30 (0.78)	3.89 (0.57)
I found the various functions in this system were well integrated	3.65 (1.19)	3.78 (0.63)
I think that I would like to use this system frequently	3.60 (0.73)	3.56 (0.68)
I needed to learn a lot of things before I could get going with this system	2.75 (1.7)	3.33 (0.94)
I think that I would need tech support to be able to use this system	1.80 (1.33)	2.67 (1.15)
I found the system very cumbersome to use	1.7 (0.84)	2.22 (1.03)
I found the system unnecessarily complex	1.65 (0.91)	2.11 (1.1)
I thought there was too much inconsistency in this system	1.40 (0.58)	1.89 (0.57)
Total	77.88 (14.02)	65.28 (12.66)

Values are presented as mean (SD).

SD = standard deviation, SUS = system usability scale.

#### 3.2.2. Understandability.

The PEMAT yielded a score of 98% from physicians. In general, physicians felt that the content of the MCA were expressed in a manner which was easy for patients to understand. Across the 11 items assessed in PEMAT, every item yielded 100% except for the item of “the material provided a summary” which had a score of 80% (Table 3). Patients surveyed through the Information quality assessment found both contents related to disease and biologics easily understandable (95% for disease; 85% for biologics).

**Table 3:** Results on understandability assessment using PEMAT; level of agreement.

Items	Agreed, n (%)
Material purpose is completely evident	10 (100.00)
Material uses common, everyday language	10 (100.00)
Medical terms are defined	10 (100.00)
Material uses the active voice	10 (100.00)
Information is provided into short sections	10 (100.00)
Sections have informative headers	10 (100.00)
Information is in a logical sequence	10 (100.00)
Visual cues to draw attention to key points	10 (100.00)
Text on the screen is easy to read	10 (100.00)
Clear and uncluttered illustrations	10 (100.00)
Material provides a summary	8 (80.00)
PEMAT score, mean (SD)	98.0 (0.04)

Values are presented as percentage (%) of participants who responded “agreed” to each question and mean PEMAT score with SD.

PEMAT = Patient Education Materials Assessment Tool, SD = standard deviation.

#### 3.2.3. Information quality assessment.

**Figure 2.**
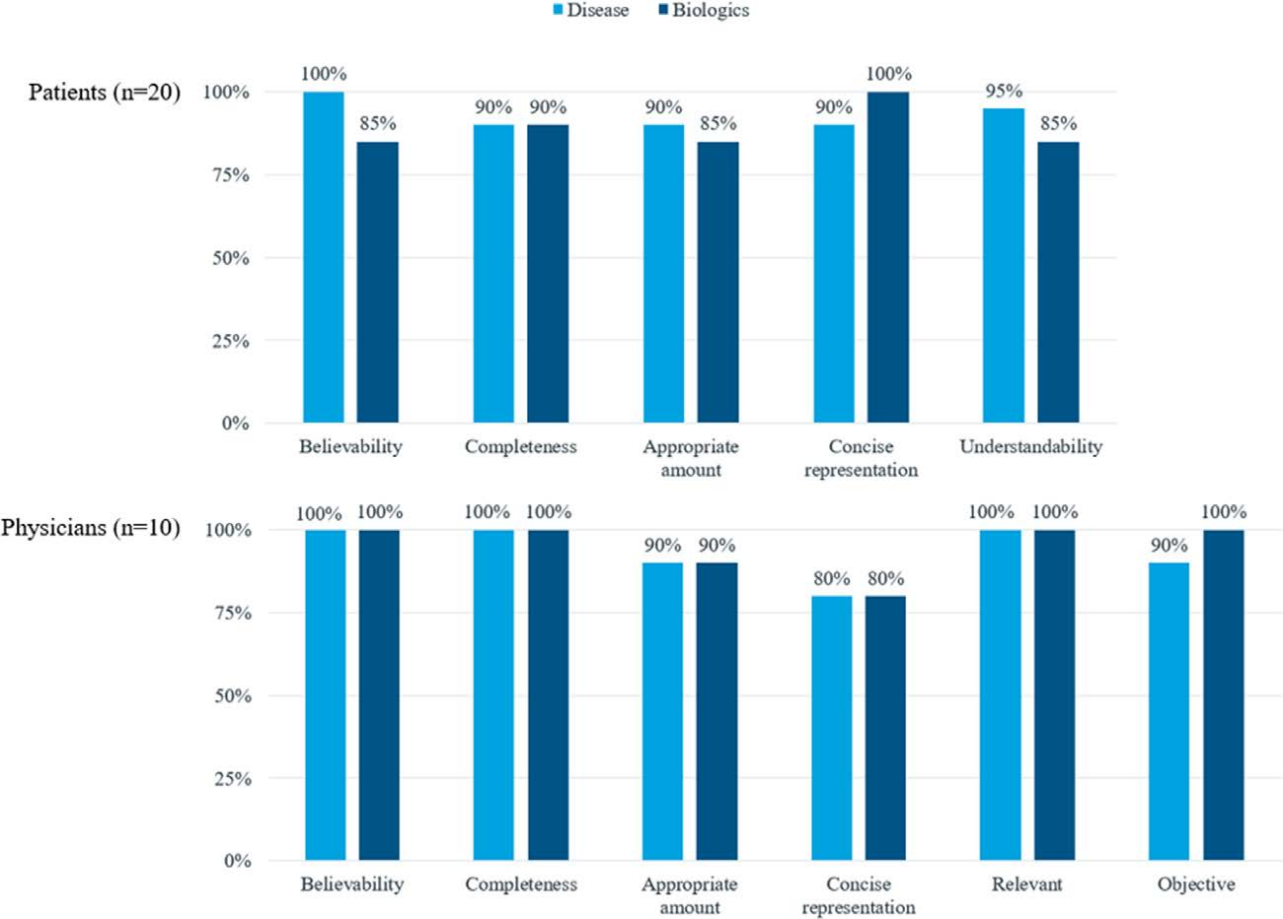
Results on information quality assessment: level of agreement. Values are presented as percentage (%) of participants who responded “agreed” to each question.

In general, most physicians and patients consistently agreed that the quality of information was high (scores above 80%) across all assessed attributes; appropriateness in the amount of text, believability, completeness, concise representation, objectivity and relevancy (Fig. 2). Yet there was a difference in the perception on quality of information related to disease vs biologics. Patients rated disease information to be of higher quality (93.0%) than biologics (89.0%). On the contrary, physicians felt that the information on biologics (95.0%) were of higher quality than disease (93.3%). Physicians gave the lowest scores for the attribute of concise representation (80%) while the patients had the lowest scores for appropriateness in the amount of text (85%).

### 3.3. Qualitative prototype testing

#### 3.3.1. Theme 1: Usefulness of the MCA.

Generally, study participants found the MCA useful and expressed a willingness to use the MCA. The responses received from patients and physicians highlighted the potential role of the MCA in fulfilling the current unmet needs in clinical care and patient education. The MCA provided easily accessible evidence-based information to the patients.

*“It’s a good educational material for patients, I would like to suggest my patients to visit the MCA website as a supplement to limited consultation time.”*

*“The MCA provides qualified and reliable information compared to other resources. I have faced some difficulties in finding good resources from the internet, social media or blogs.”*

#### 3.3.2. Theme 2: Content of the MCA.

The content of the MCA was developed mainly in the native Korean language. While the understandability score of the MCA were high, few patients expressed difficulties in understanding the content due to their unfamiliarity with some terminologies presented in English. The Others felt that the current contents, especially on biologics, were difficult to understand given their limited existing knowledge on this topic.

*“It would have been easy to understand if it was not in English”*

*“Because I do not have any background knowledge or experience, biologics part was hard to understand”*

Overall, physicians felt that the current MCA contents are well expressed and organized which helps improve the overall understandability of what could be a difficult topic. However, few physicians disagreed with the amount of information presented and thought that content was not concise enough for patients.

*“Expressions are not simple or concise enough”*

*“No need to provide all information to everyone, it would be better to separate the users by AS severity”*

Participants also provided feedbacks with regards to the additional contents which they would like to see in the MCA. Patients would like to have more detailed information related to lifestyle management such as exercise, diet, and personal attitude. Physicians would like to emphasize the need for patients to consult their specialists prior to making any changes related to their treatment.

*“I need examples of food to be avoided.”*

*“Medication should not be discontinued by themselves, particularly when the side effects occur”*

#### 3.3.3. Theme 3: Design of the MCA.

While the usability score was above average, study participants identified several limitations with the design of the MCA. It was thought that the cover page of the MCA was not intuitive enough to allow easy navigation through its content. Patients had difficulties navigating through the MCA to find the right information.

*“I might be lost in finding the right information in the current format”*

*“It would be great to see all questions in the same page”*

## 4. Discussion

In this study, we included the perspectives of both patients and physicians in assessing the usability and understandability of the MCA. The MCA was developed to support shared decision-makings between physicians and AS patients through improved understanding of patients’ knowledge and preferences. Findings from the study suggested that the MCA was usable and highly understandable. Through the study, we identified several limitations of the MCA which would be used to guide further refinement of the MCA prototype. Importantly, patients and physicians found the information provided in the MCA useful and had expressed willingness to continue using the tool.

To our knowledge, this was the first interactive web-based MCA designed for AS in Korea. Similar patient decision aids have been developed in other therapeutic areas such as oncology and neurology.^[26–28]^ Previous studies mainly focused on evaluating the impacts of these decision aids on health outcomes and decision-makings.^[26–28]^ Hence, we were not able to make comparisons between the results from our usability testing with evaluations of previously developed decision aids.

There were several strengths to our study. The results of our study were bolstered by a mixed-methods research. The in-depth interviews with embedded think-aloud approach allowed a more comprehensive understanding of the survey responses on usability and understandability. We managed to recruit a total of 10 and 20 study participants for the patient and physician subgroups respectively. This sample size was deemed optimal to identify at least 80% of usability issues as evident by the saturation of themes observed in the results. The profiles of our study participants were considered diverse in terms of patients’ age and disease duration, as well as physicians’ years of clinical experience. Findings from each item assessed were mainly consistent between patients and physicians. We observed the only difference found between the two groups was the perception on quality of information related to disease *vs*. biologics. Patients rated the quality of disease information better than biologics while physicians felt that the information on biologics were of higher quality than disease. This disagreement in quality perception could be driven by the limited knowledge and understanding on biologics of the patients as they were naïve to biologic treatments.

Several limitations should be considered when interpretating our results. Firstly, the MCA was developed for biologics naïve patients. Hence, our study results may not apply to patients who are already on biologics or considering a switch of biologics due to the progressive nature of AS. Additionally, the study was conducted mainly with study participants receiving or providing care at general or tertiary hospitals within the urban areas of South Korea. Assessments on usability and understandability of the MCA may not be generalizable to populations in other regions specifically in the rural areas where clinical pathways and treatment options may differ.

Our study highlighted the current unmet needs and limitations with regards to medical communication in AS. In the present context, physicians often have limitation in patient education and interaction given their high patient load and limited resources. The MCA could enhance the patient-physician communication by providing information on disease and treatment options. More consultation time could then be spent addressing patients’ other needs and concerns. The MCA helped increase patient awareness and knowledge to participate in decision-making, leading to improved healthcare outcomes. The promotion of interactive patient-physician partnership in decision-making is particularly relevant for chronic conditions where patient’s self-management and involvement are considered a major part of treatment journey. Previous studies have also shown that a good MCA could reduce unnecessary medical resource expenditures and contribute to quality enhancement of the public health care.^[29–31]^ This led to the recommendation that the use of patient decision aids should be promoted and implemented by a public institution such as the Korean National Evidence-based Health Care Collaborating Agency.^[32]^ Despite some limitations with the MCA, our study found that physicians were mostly willing to use the tool. Under the Korean national health insurance, a fee-for-service reimbursement system is utilized where providers receive payment for each service rendered.^[33]^ To encourage the uptake of patient education, policymakers could explore the possibility of developing reimbursement schemes for these preventive health services. The reimbursement might incentivize physicians to perform all the necessary tasks (including patient education) in delivering comprehensive AS care.

The introduction of the MCA would affect the clinical management of AS patients and the daily workflow of healthcare professionals. If the MCA was to be rolled out officially, a feasibility assessment would be required during the pre-implementation phase to enable smooth integration of the MCA into routine clinical practice.^[34]^ The MCA would need to be tailored to different clinical settings and consider different patient preferences for AS treatment. Future research can focus on assessing the impact of a validated MCA in supporting shared decision-making and the quality of decisions made.

## 5. Conclusion

We have developed a usable and highly understandable web-based communication aid to support shared-decision making between patients and physicians. Our study identified several limitations with the existing prototype which would guide further enhancement of the communication aid. The MCA had great potential in supporting shared decision-making by improving patients’ knowledge on the disease and available treatment options, as well as clarifying patients’ personal preferences and values in the management of AS.

## Author contributions

**Conceptualization:** Sang-Hoon Lee, YoungJu Park, Chan-Bum Choi, Yong-Gil Kim, Jung-Ae Kim, Hoon-suk Cha.

**Formal analysis:** Sang-Hoon Lee, YoungJu Park, Jung-Ae Kim.

**Methodology:** Sang-Hoon Lee, YoungJu Park, Chan-Bum Choi, Yong-Gil Kim, Jung-Ae Kim, Hoon-suk Cha.

**Project administration:** Jung-Ae Kim.

**Supervision:** Sang-Hoon Lee, YoungJu Park, Jung-Ae Kim, Hoon-suk Cha.

**Validation:** Sang-Hoon Lee, YoungJu Park, Jung-Ae Kim, Hoon-suk Cha.

**Writing – original draft:** Sang-Hoon Lee, YoungJu Park, Jung-Ae Kim, Hoon-suk Cha.

**Writing – review & editing:** Sang-Hoon Lee, YoungJu Park, Chan-Bum Choi, Yong-Gil Kim, Jung-Ae Kim, Hoon-suk Cha.
